# Measurements of dune erosion processes during the RealDune/REFLEX experiments

**DOI:** 10.1038/s41597-024-03156-9

**Published:** 2024-04-23

**Authors:** Paul van Wiechen, Jantien Rutten, Sierd de Vries, Marion Tissier, Ryan Mieras, Katherine Anarde, Christine Baker, Ad Reniers, Jan-Willem Mol

**Affiliations:** 1 Department of Hydraulic Engineering, Delft University of Technology, Stevinweg 1, 2628 CN Delft, The Netherlands; 2 Department of Physics and Physical Oceanography, University of North Carolina Wilmington, 601 South College Road, Wilmington, NC 28403 USA; 3 North Carolina State University, 915 Partners Way, Raleigh, NC 27695 USA; 4 Dutch Ministry of Infrastructure and Water Management, Griffioenlaan 2, 3526 LA Utrecht, The Netherlands; 5 Present Address: Department of Resilient Coasts and Ports, Unit of Hydraulic Engineering, Deltares, 2629 HV Delft, The Netherlands

**Keywords:** Physical oceanography, Natural hazards

## Abstract

Nearshore hydro- and morphodynamic data were collected during a field experiment under calm conditions, moderate conditions, and storm conditions with dune erosion in the collision regime. The experiment was conducted on the Sand Engine near Kijkduin, the Netherlands, from October 18, 2021, to January 7, 2022. Two artificial unvegetated dunes were constructed just above the high water line to measure storm erosion and dune impacts from higher water levels and waves. During the experiment, three storms occurred that resulted in significant erosion of both dunes. The collected hydrodynamic data include pressure sensor and velocimeter data along two cross-shore transects. The collected morphodynamic data include bathymetry and topography surveys, optical backscatter sensor data in the inner surf zone, and a continuous cross-shore line-scanning lidar data set of the dune face. This comprehensive data set can be used to (1) study relevant nearshore hydrodynamic and morphodynamic processes that occur during calm conditions, moderate conditions, and storm conditions with dune erosion in the collision regime, and (2) validate existing dune erosion models.

## Background & Summary

At sandy coastlines, storm conditions can lead to substantial erosion of the dunes with the risk of flooding of the hinterland^1–3^. Moreover, the current rate of sea level rise and climate change further endangers our global coastlines and the people living in areas protected by dunes^4,5^. To continuously assess the risks these sandy coastlines face, researchers and engineers often rely on existing knowledge and dune erosion models to predict storm impact^6–9^. However, not all processes that occur during dune erosion are fully understood, which translates to uncertainties in model predictions^10,11^. New insights into these processes can be acquired by analysing field data. Field experiments therefore prove a valuable resource to understand the behaviour of dunes and to increase our coastal resilience.

**Fig. 1:**
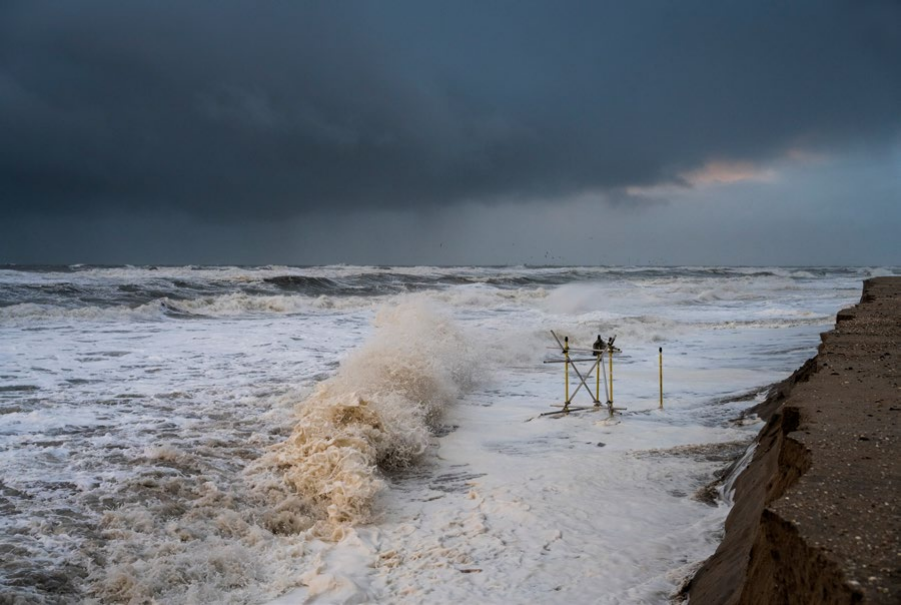
Impression of dune erosion at Dune 1 of the field site (Fig. 2) during storm conditions on January 5, 2022 (Ph. Mischa Keijser). Instruments were attached to poles and frames in front of the dunes. A 2DV lidar scanner was installed on the frame in this photograph.

This paper presents nearshore hydro- and morphodynamic data that were collected during the RealDune/REFLEX field experiments along the Dutch coast in the 2021 autumn and 2021/2022 winter, designed to investigate dune erosion in the field. The specific focus here is on the data collected in the nearshore around two artificial, unvegetated dunes of 5.5 m high and 150 m wide between October 18, 2021, and January 7, 2022 (Fig. 1). Within the RealDune/REFLEX experiments, high-resolution data were also collected from November 2021 to April 2022 at several offshore locations in intermediate water depth. These continuous measurements describe the offshore hydrodynamic conditions and wave transformation in the North Sea over a longer period of five months. More details of these offshore measurements are described in the accompanying paper by Rutten *et al*.^12^.

**Fig. 2:**
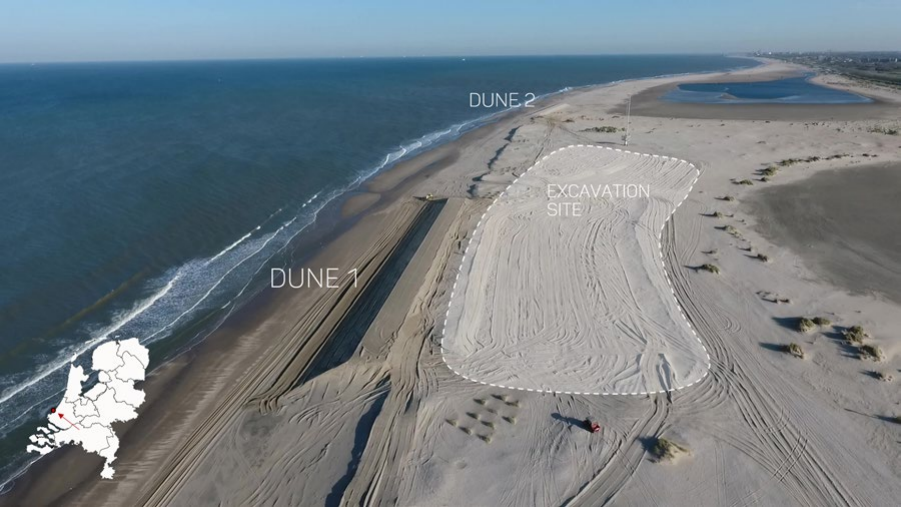
Aerial view of the field site including Dune 1, Dune 2, and the excavation site from which sediment was excavated for the construction of both dunes. The field site location within the Netherlands is displayed on the inset map.

The nearshore data described here were collected on the Sand Engine (The Netherlands), an artificial peninsula between Kijkduin and Monster that serves as a mega-nourishment for the surrounding coasts^13^ (Fig. 2). Two dunes were constructed at separate locations with differences in coastline orientation and subtidal bathymetry^14–16^ (Fig. 2). As a consequence, identical offshore wave conditions were assumed to result in different nearshore wave conditions at each of the two dunes, which doubled the data to be used for analysis of specific processes. Both dunes were constructed shoreward of the 1.5 m NAP (Normaal Amsterdams Peil, the Dutch coordinate system) depth contour, being just above, and in close proximity to, the high water line. This increased the probability of dune erosion events occurring during the experiment. In the end, three storms occurred that resulted in erosion of the dune face. The largest of the three storms occurred on January 5, 2022, and had a total mean water level with a return period of approximately 1-2 years^17^. Overall, throughout the experiment, significant wave heights recorded at the most seaward nearshore station ranged from 0 to 2 m, and surge contributions to the total mean water level ranged from 0 to 1.2 m. This resulted in calm conditions, moderate conditions, and storm conditions with dune erosion in the collision regime. Altogether, the data set can be used to (1) study the relevant nearshore hydrodynamic and morphodynamic processes that occur during calm, moderate, and storm conditions with dune erosion in the collision regime, and (2) validate existing dune erosion models.

## Method

### Site and design dunes

The field site was located on the Sand Engine near Kijkduin, the Netherlands (Fig. 2). The southern dune is defined as Dune 1. The northern dune, located at the tip of the Sand Engine, is defined as Dune 2. Both dunes were constructed from October 18 to October 27. Sediment from the upper layer (∼1 m) from the higher region of the Sand Engine was excavated using a crane, then transported using dumpers, and finally shaped into dunes by a bulldozer. The dumpers and bulldozer continuously drove over the deposited sediment during the two week construction period to increase the compaction of the sediment. However, data about the compaction and porosity are not available for this data set. The excavated sediment was sieved by the laboratory facilities of the University of Utrecht. The sediment is slightly gravelly sand, with a gravel content of 0.2% and sand content of 99.8%. The sediment had a *D*_10_, *D*_25_, *D*_50_, *D*_75_, and *D*_90_ of respectively 236.0, 287.3, 362.3, 443.0, and 557.6 *μ*m.

**Fig. 3:**
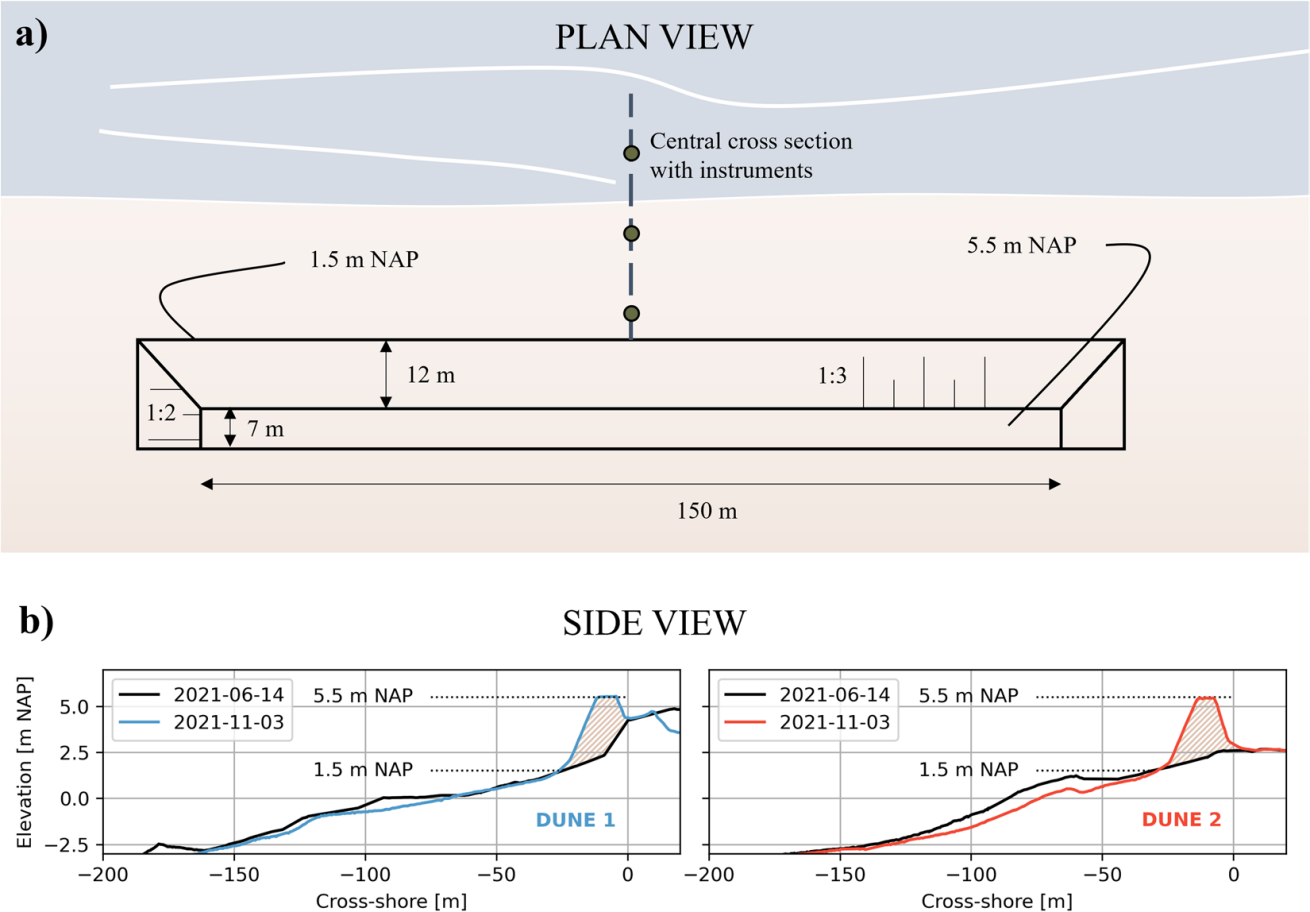
(**a**) Plan view of the constructed dunes with dune dimensions. The dimensions are identical for both dunes. (**b**) Side view of the central cross-section before (black) and after construction of Dune 1 (blue in left panel) and Dune 2 (red in right panel).

The initial dune toes of both dunes were located at the +1.5 m NAP depth contour (Fig. 3). The crests were at +5.5 m NAP. The initial slopes of the dune faces were 1:3, resulting in dune faces of 12 m wide in cross-shore direction. The dune crests were 7 m wide in cross-shore direction, totalling the cross-shore width of the dunes to 19 m. Behind the dune crests, the profiles run back to the original beach under a slope of 1:2. The lengths of the dunes in alongshore direction were 150 m. The cross-shore volume of the dunes was approximately 68 m^3^/m, depending on the original beach profile below the constructed dune. The total volume of excavated sediment required for construction of both dunes was approximately 20000 m^3^.

The slope of the dune face of both dunes was based on a representative dune profile of the Holland coast in the Netherlands^18^. This profile was also used in laboratory flume tests for the development of dune erosion models and dune safety assessments in the Netherlands^7,8,18,19^. The location and vertical elevation of the dune toe of both dunes was based on the return periods of mean water levels of storm surges in the region, consisting of tide, wind setup and surge. The requirement was that the mean water level of a storm with a 0.1 year return period should exceed the dune toe, resulting in measurable erosion. The cross-shore length of both dunes was based on the criterion that they should contain enough sediment to withstand a storm with a 2 year return period. The storm characteristics and return periods were based on wave data from an offshore wave platform in close proximity to the field site (Europlatform wave platform). The total erosion of the dunes induced by the storms with a 0.1 year and 2 year return period were assessed using XBeach two-dimensional horizontal (2DH) in surfbeat mode^9^.

The height of the crest (+5.5 m NAP) ensured that the dunes would remain in the collision regime^20^ during a moderate storm surge. Lastly, the alongshore width of both dunes was based on the criterion that the alongshore currents, generated by wave breaking, would be fully developed in the central 50 metres of each dune, and that the edge effects of the dune edges would become negligible in this central segment. This criterion was also assessed using the 2DH XBeach model.

### Deployments and instrumentation

**Fig. 4:**
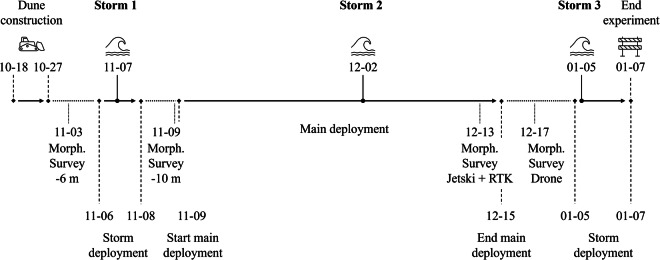
Timeline of the experiment consisting of two storm deployments and one main and more detailed deployment. Three storms occurred during the experiment. Morph. Survey −6 m stands for a survey of the topography and bathymetry up to the −6 m NAP depth contour. Survey −10 m stands for a bathymetric survey from the −6 m to −10 m depth contour. The morphological survey of December 13 contains the inter- and subtidal bathymetry which was recorded with a jetski and an RTK GPS walking survey. The morphological survey of December 17 contains the topography which was recorded with a drone.

The experiment can be divided into an initial storm deployment from November 6 to November 8, a main and more detailed deployment from November 9 to December 15, and another storm deployment from January 5 to January 7 (Fig. 4). Storms occurred on November 7, December 2, and January 5. Dune 2 moved from the collision regime into the overwash regime^20^ during the January 5 storm, and was completely eroded after the storm.

#### The initial November storm deployment

**Fig. 5:**
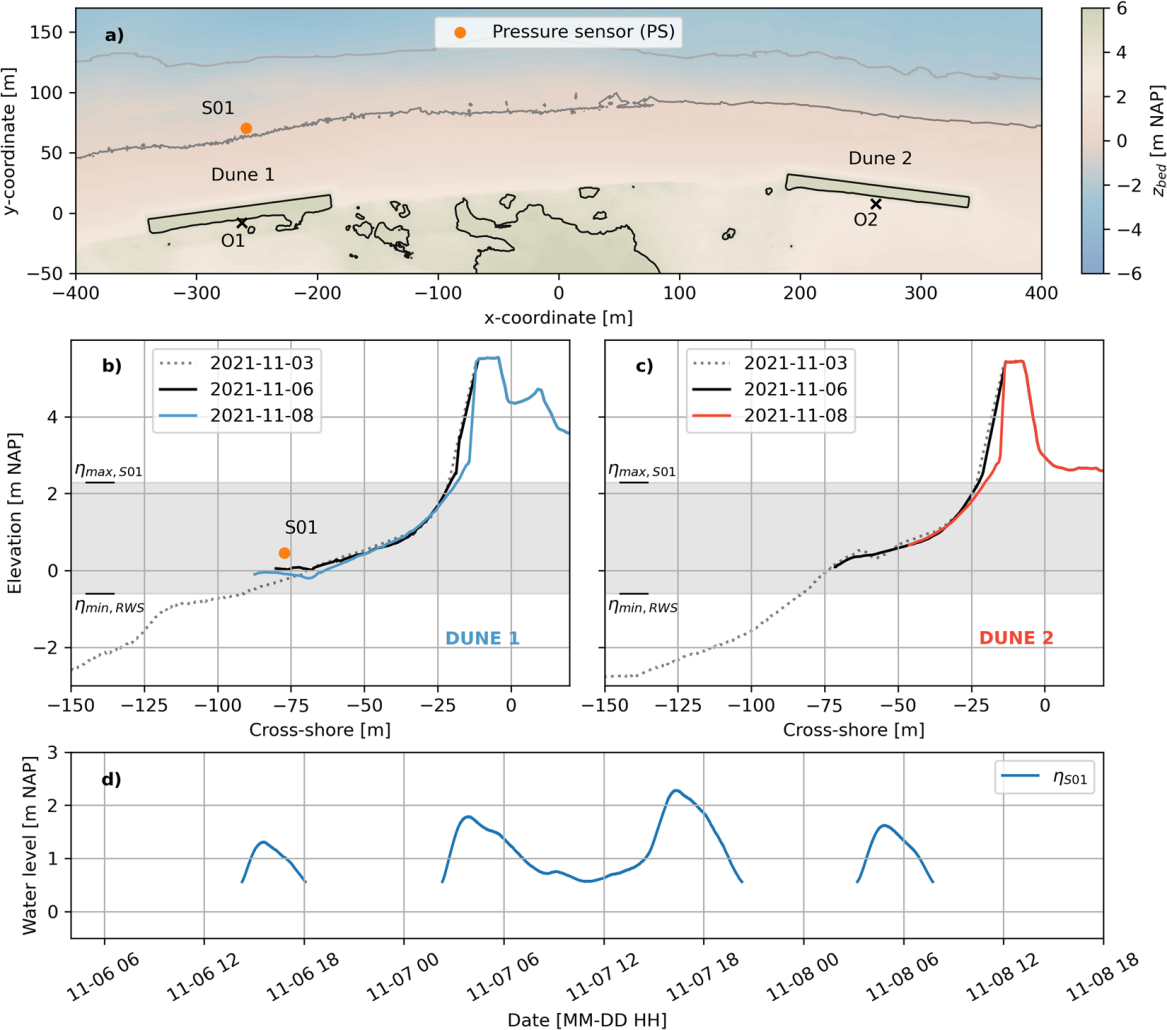
Plan view of both dunes, including the deployed pressure sensor (**a**), and cross-shore profiles of Dune 1 (**b**) and Dune 2 (**c**), during the initial November storm deployment. Panel (**d**) displays the 30-minute average water levels recorded at S01 during the deployment. In (**a**), O1 and O2 represent the origins of the cross-shore - longshore coordinate systems of both dunes, whereas the x- and y-coordinates are with respect to a temporary coordinate system that holds no meaning in the remainder of this paper and is for illustrative purposes only. In panels (**b**) and (**c**), *η*_max, S01_ represents the maximum water level of (**d**), and *η*_min, RWS_ represents an estimation of the minimum mean water level at the field site during the deployment, based on water level measurements conducted by Rijkswaterstaat (The Dutch Ministry of Infrastructure and Water Management).

The initial November storm deployment covered four consecutive high tides, of which the third high tide was largest in magnitude with a peak water level of 2.28 m NAP on November 7 16:19 local time (Fig. 5). All times hereafter are given in local time unless stated otherwise. A bathymetric and topographic survey to the −6 m NAP depth contour was conducted on November 3, using a jetski, an RTK GPS walking survey, and a drone. A pre-storm RTK GPS^21^ walking survey was conducted on November 6 and a post-storm survey was conducted on November 8. Only one pressure sensor (PS), S01_OSSI_01 (OSSI wave gauge^22^), was deployed, which was located in close proximity to Dune 1 (Fig. 2). S01_OSSI_01 had a sampling frequency of 10 Hz and recorded at a constant elevation of +0.46 m NAP during the deployment. The initial bed was 0.306 m below this sensor. The height of the instrument above the bed after the storm can be computed using the post-storm RTK GPS survey and the fixed elevation of the sensor at +0.46 m NAP.

#### The main November - December deployment

**Fig. 6:**
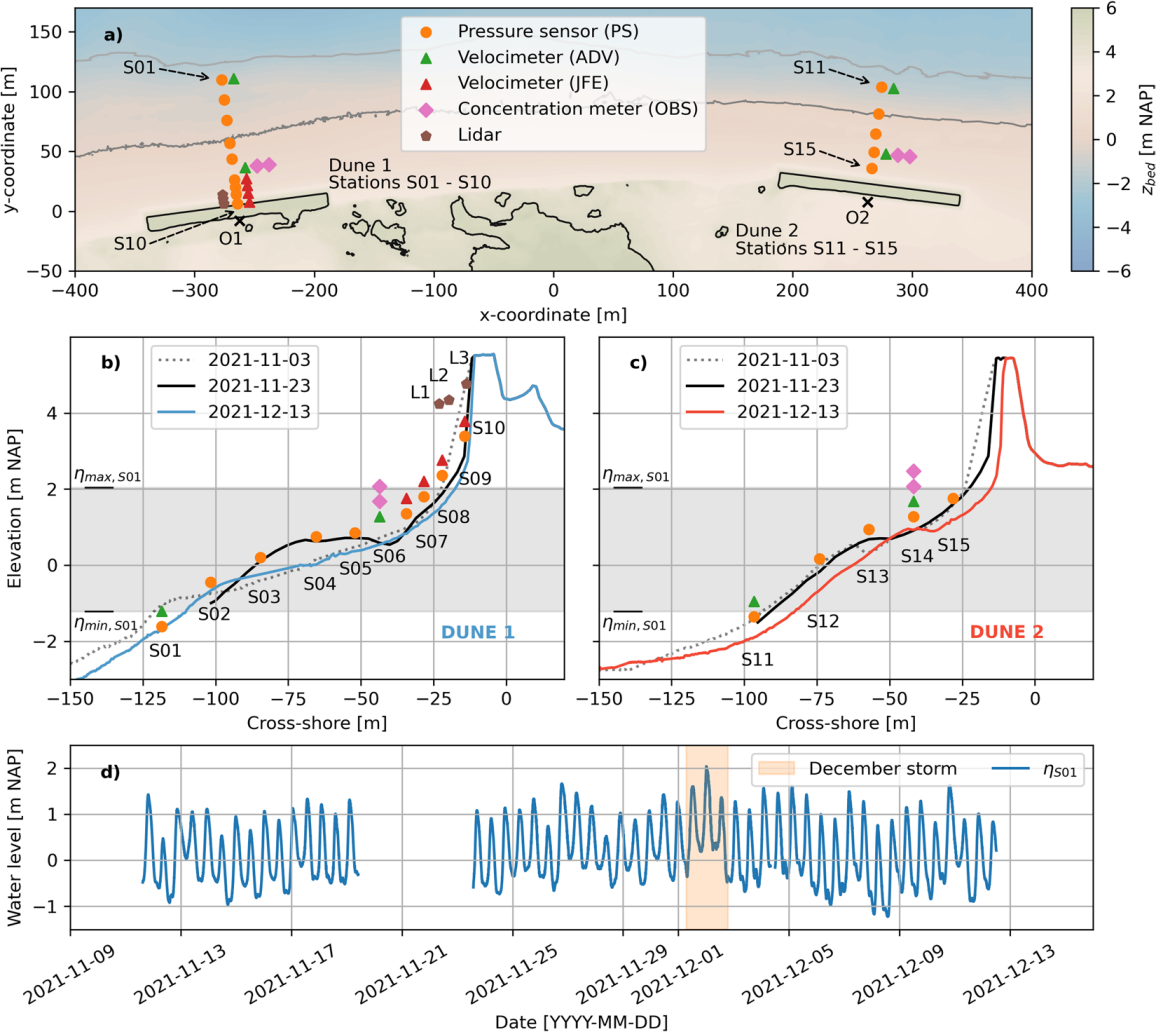
Plan view of instrumentation at both dunes (**a**), and cross-shore instrumentation of Dune 1 (**b**) and Dune 2 (**c**), during the main November-December deployment. Panel (**d**) displays the 30-minute average water levels recorded at S01 during the main deployment. The December storm surge occurred from December 1 to December 2 (orange). O1 and O2 in (**a**) represent the origins of the cross-shore - longshore coordinate systems of both dunes. The x- and y-coordinates in (**a**) are with respect to a temporary coordinate system that holds no meaning in the remainder of this paper and is for illustrative purposes only. The elevations of the instruments in panels (**b**) and (**c**) have been changed vertically for readability and are not exactly equal to their elevations in the field. S06 had no standalone pressure sensor. Here, pressure was recorded by the built-in pressure sensor of the ADV. In panels (**b**) and (**c**), *η*_max, S01_ and *η*_min, S01_ represent the maximum and minimum water levels of (**d**).

The detailed main deployment lasted from November 9 to December 15. Within this period, a significant storm surge passed from December 1 to December 2, with a peak water level of 2.04 m NAP on December 2 00:20 (Fig. 6).

A bathymetric survey from the −6 to −10 m NAP depth contour was conducted with a jetski on November 9. A bathymetric survey of the sub- and intertidal area up to the −10 m NAP depth contour was conducted with a jetski and an RTK GPS walking survey on December 13. A topographic survey of the supratidal area was conducted with a drone on December 17. RTK GPS walking surveys of the intertidal zone were conducted on November 15, 22, 27 and 30, and December 1, 2 and 8. The surveys differ in the area covered. The central cross-section of both dunes was recorded on November 11, November 15 to November 20, November 23 to December 10, and December 12. This central cross-section runs from the deepest point offshore that was accessible with the RTK GPS towards the dune toe. Profile information of the central cross-sections above the dune toe can be acquired using the walking and drone surveys.

**Fig. 7:**
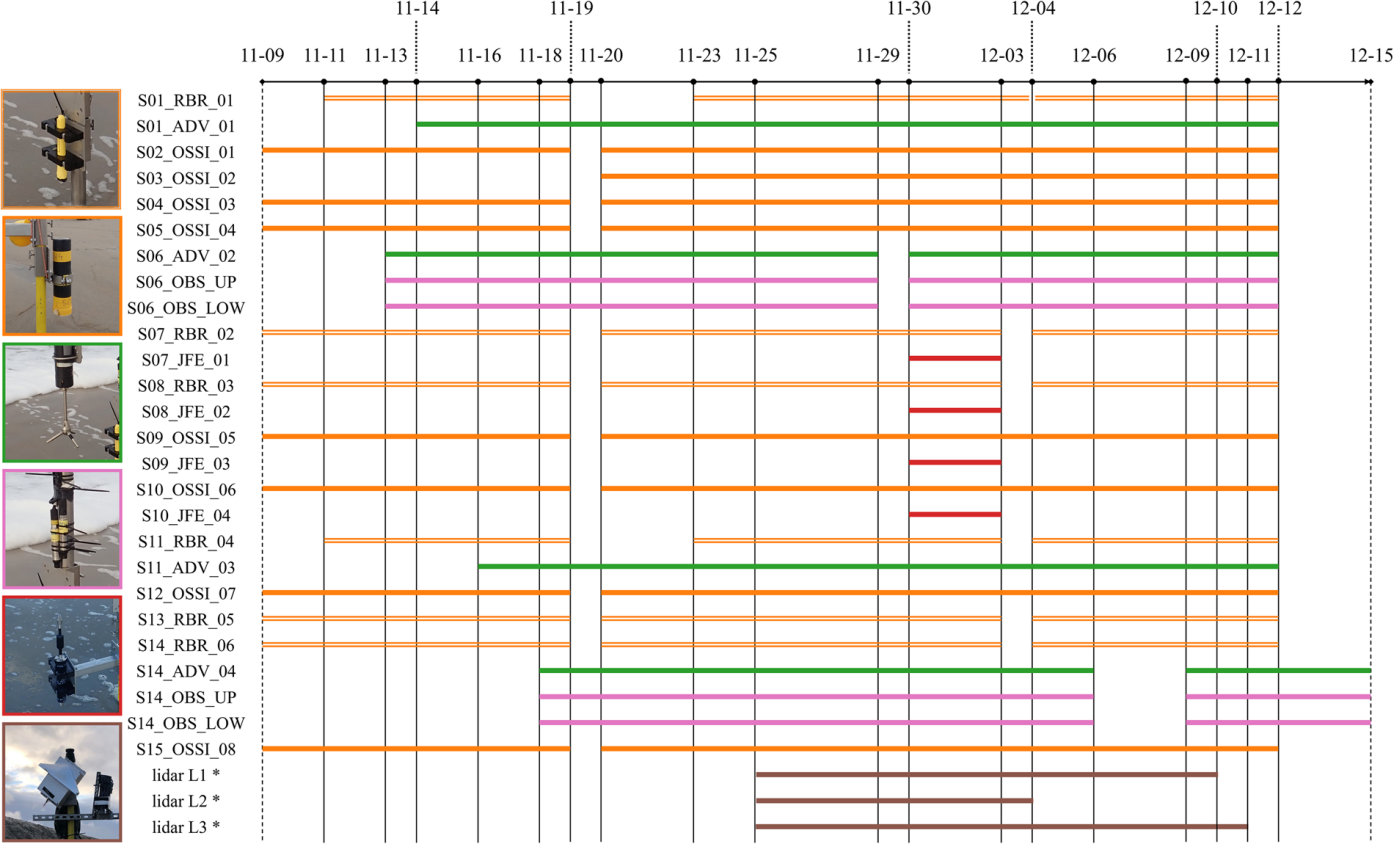
Deployment times during the main November-December deployment of the pressure sensors (RBRs open orange, OSSIs filled orange), velocimeters (ADVs green, JFEs red), OBSs (pink), and lidars (brown). Gaps in each timeline represent periods in which the instrument was not in the field, most likely due to a service interval in which batteries of the instrument were replaced and data were retrieved. * The lidars did not always produce data when they were in the field, most likely because the intensity of the reflected beam was insufficient, or due to power supply issues. This means there are not always processed data files for the times displayed in the timeline above. e.g., lidar 2 (L2) was unable to produce data during the December 2 storm.

At Dune 1, nine pressure sensors (PS, OSSI wave gauges^22^ and RBR solo pressure sensors^23^), two 3D velocimeters (ADV, Nortek Vector Accoustic Doppler Velocimeters^24^), two optical backscatter sensors (OBS, Campbell OBS3+^25^), and four 2DH velocimeters (JFE Advantech Infinity electromagnetic current meters^26^) were installed at ten different stations (Fig. 6). The JFEs were only installed during the December 2 storm surge. In addition, three standalone cross-shore Line-Scanning, Low-Cost (LLC) 2DV lidar systems^27^ were placed on the upper beach at Dune 1 to record the evolution of the dune profile (L1, L2 and L3). At Dune 2, five pressure sensors, two velocimeters, and two OBSs were installed at five different stations. RBR solo wave gauges were installed at stations S01, S07, S08, S11, S13 and S14, and recorded pressure with a sampling frequency of 8 Hz. OSSI wave gauges were installed at stations S02, S03, S04, S05, S09, S10, S12, and S15, and recorded pressure with a sampling frequency of 10 Hz. S06 had no standalone pressure sensor. Here, pressure was recorded by the built-in pressure sensor of the ADV. The ADV velocimeters recorded velocity and pressure at 16 Hz. The OBSs recorded backscatter at 16 Hz. The JFE 2DH velocimeters recorded voltage (induced by water flowing through an electromagnetic field) at 1 Hz. The lidars collected data in 35-minute bursts at the top of each hour with approximately 6 rotations per second. The angular resolution within a single rotation was approximately 0.3 degrees; however, angular resolution is not consistent due to the low cost nature of the lidar range scanner (USD110). This resulted in slight variations in the angle range of the measurements of each rotation, yielding an overall angular resolution higher than 0.3 degrees. Time intervals during which each instrument was deployed are displayed in Fig. 7.

The instrument naming for the RBRs, OSSIs, ADVs, and JFEs in the main November-December deployment data files is *SXX_YYYY_ZZ*, where SXX denotes the station number at which the instrument was deployed (e.g. S08), YYYY the instrument type (e.g. RBR), and ZZ the instrument number (e.g. 03). The OBSs are named by the station number, with an indication whether it is the upper or lower OBS at that station. The lidars are called L1, L2 and L3, where L1 is the most seaward lidar and L3 the most landward lidar (Fig. 6).

The instruments were moved upwards or downwards along the pole to which they were attached to retain near-bed measurements given changing bathymetry of the field site during the experiment. On- and offshore intertidal bar migration and/or morphologic resets^28^ resulted in a highly variable bed. Instruments were moved downwards when the bed level decreased in height to keep them close to the bed, and thus, ensuring that measurements were recorded for the longest possible time period given varying water levels. Instruments were moved upwards when the bed level increased in height to avoid instrument burial. The elevations of the instruments with respect to the bed were daily recorded and documented, if the hydrodynamic conditions allowed. Note that the elevation measurements started on November 13. Instruments already in the field before November 13 (pressure sensors, Fig. 7) remained at the same vertical position in the days before November 13. When the instruments were moved vertically along the pole to which they were mounted, the old and new position with respect to the bed were documented. Periodically, the instrument location was recorded with the RTK GPS so that the instrument could be placed in the local frame of reference.

The beach and dune face of Dune 1 were observed by two infra-red (IR) cameras from November 30 to December 4. The IR cameras were installed near the dune face of Dune 1 and collected one frame every minute. One camera was facing towards the dune, the other seawards. In addition to the two IR cameras, two GoPro cameras were installed during the peak of the December storm near the dune face of Dune 1 from December 1 23:20 to December 2 4:00. Both GoPros faced the dune face and collected two frames every second.

#### The January storm deployment

**Fig. 8:**
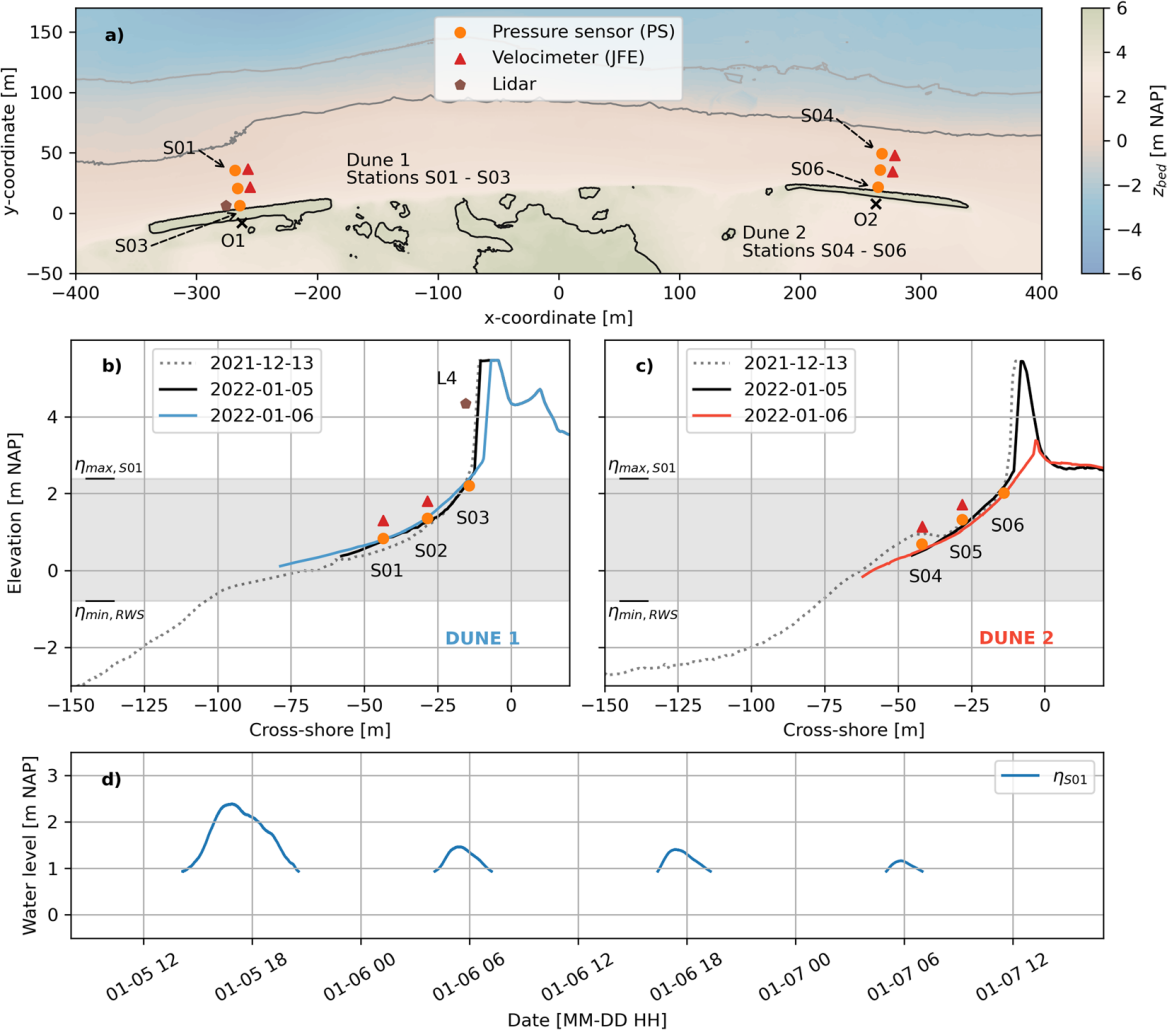
Plan view of instrumentation at both dunes (**a**), and cross-shore instrumentation of Dune 1 (**b**) and Dune 2 (**c**), during the January storm deployment. Panel (**d**) displays the 30-minute average water levels recorded at S01. In (**a**), O1 and O2 represent the origins of the cross-shore - longshore coordinate systems of both dunes, whereas the x- and y-coordinates are with respect to a temporary coordinate system that holds no meaning in the remainder of this paper and is for illustrative purposes only. The elevations of the instruments in panels (**b**) and (**c**) have been changed vertically for readability and are not exactly equal to their elevations in the field. In panels (**b**) and (**c**), *η*_max, S01_ represents the maximum water level of (**d**), and *η*_max, RWS_ represents an estimation of the minimum mean water level at the field site during the deployment, based on water level measurements conducted by Rijkswaterstaat (The Dutch Ministry of Infrastructure and Water Management).

The January storm deployment covered four consecutive high waters, of which the first high water was largest in magnitude with a peak water level of 2.38 m NAP on January 5 16:53 (Fig. 8). A pre-storm RTK GPS walking survey was conducted on January 5 and a post-storm survey was conducted on January 6. The central cross-section of both dunes was recorded on January 5, 6 and 7.

During the January storm deployment, three pressure sensors (RBR solo pressure sensor) and two 2DH velocimeters (JFE Advantech Infinity electromagnetic current meters) were deployed at each dune (Fig. 8). The pressure sensors had a sampling frequency of 8 Hz. The JFEs had a sampling frequency of 10 Hz. One LLC lidar scanner was deployed near the dune face at Dune 1 that recorded the evolution of the dune profile during the storm (L4). Data were collected in 50-minute bursts at the top of each hour with the same average sampling frequency (∼6 rotations per second) and angular resolution (∼0.3 degrees) as the lidars deployed during the main November - December deployment.

The instrument naming for the January storm deployment is *SXX_YYYY_ZZ*, where SXX denotes the station number at which the instrument was deployed (e.g. S05), YYYY the instrument type (e.g. JFE), and ZZ the instrument number (e.g. 04). The lidar is labelled L4.

## Data Records

All data were stored with open access on the 4TU.ResearchData repository^29^. The data are stored as a data collection with name ‘Nearshore coastal measurements of calm, moderate, and storm conditions at two artificial dunes along the Dutch Coast during the RealDune/REFLEX experiments’, with 10.4121/0a05d041-00b6-4e8e-a5c5-70e624ea720b.

The data collection includes 11 data sets:

- 20211103 20211109 Bathy Topo Survey Shore Monitoring - Nearshore - RealDune/REFLEX
- 20211106 20211108 Storm deployment - Nearshore - RealDune/REFLEX
- 20211109 20211215 Main deployment - GoPro 1 - Nearshore - RealDune/REFLEX
- 20211109 20211215 Main deployment - GoPro 2 - Nearshore - RealDune/REFLEX
- 20211109 20211215 Main deployment - lidar 1 - Nearshore - RealDune/REFLEX
- 20211109 20211215 Main deployment - lidar 2 - Nearshore - RealDune/REFLEX
- 20211109 20211215 Main deployment - lidar 3 - Nearshore - RealDune/REFLEX
- 20211109 20211215 Main deployment - Nearshore - RealDune/REFLEX
- 20211213 20211217 Bathy Topo Survey Shore Monitoring - Nearshore - RealDune/REFLEX
- 20220105 20220107 Storm deployment - Nearshore - RealDune/REFLEX
- Supplementary data - Nearshore - RealDune/REFLEX

The GoPro images and lidar data of the main November - December deployment have been stored as separate data sets because of the relatively large size of the data. All other data of the main November-December deployment have been stored in *20211109 20211215 Main deployment - Nearshore - RealDune/REFLEX*. The data set *Supplementary data - Nearshore - RealDune/REFLEX* contains records with the air pressure measured at the Hoek van Holland station of the KNMI (Royal Netherlands Meteorological Institute), and water levels recorded at Hoek van Holland and Scheveningen by Rijkswaterstaat (The Dutch Ministry of Infrastructure and Water Management).

### Local coordinate systems in metadata

A local cross-shore - longshore coordinate system has been defined for both dunes. The origin of the local system of Dune 1 has coordinates (72314.8 m, 451899 m) (RDNAP coordinate system of reference). The positive cross-shore axis of Dune 1 has a direction of 116° true North and the positive alongshore axis has a direction of 26° true North. The origin of the local system of Dune 2 has coordinates (72587.6 m, 452348 m). The positive cross-shore axis of Dune 2 has a direction of 130° true North and the positive alongshore axis has a direction of 40° true North. The ADV and JFE data sets contain velocities with respect to the instrument axis, with respect to the easting and northing axes of the RDNAP coordinate system, and with respect to the cross-shore and alongshore axes of the local coordinate systems. The lidar data sets contain coordinates in the RDNAP coordinate system and the local cross-shore - alongshore coordinate system.

### Detailed bathymetric and topographic surveys

The bathymetric surveys to the −10 m NAP depth contour and topographic surveys were conducted by the Dutch private company Shore Monitoring & Research. There is a data set for the November survey (*20211103 20211109 Bathy Topo Survey Shore Monitoring - Nearshore - RealDune/REFLEX*) and a data set for the December survey (*20211213 20211217 Bathy Topo Survey Shore Monitoring - Nearshore - RealDune/REFLEX*). Within each data set is the report, a pts file with point coordinates of the bathymetric survey conducted with a jetski and an RTK GPS, and a .TIF file with point coordinates of the topographic survey conducted with a drone.

For the November survey, all coordinates within the points file that are above the −6 m NAP depth contour were recorded on November 3. All coordinates between the −6 and −10 m NAP depth contours were recorded on November 9. For the December survey, all data within the.pts file were recorded on December 13. The data in the .TIF file were recorded on December 17.

The .TIF file of the December 17 drone survey includes point measurements of the poles and frames that were already installed for the January storm deployment (Fig. 8). As a consequence, these poles and frames can be observed in the data from the topographic survey. If one wants to remove these points from the .TIF file, one can use the instrument and lidar frame coordinates in the GPS instrument file of January 6, 2022 (*20220106 INSTRUMENTS.txt*).

### The initial November storm deployment

All data of the initial November storm deployment can be found in the *20211106 20211108 Storm deployment - Nearshore - RealDune/REFLEX* data set. The initial November storm deployment contains GPS data and PS data. The GPS data are divided into a folder for each day, with notation YYYYMMDD. The PS data are gathered in a netcdf file (*20211106 1000 20211108 1300 ossi.nc*). For both the GPS and PS data, the raw instrument files are also provided. Photographs for an impression of the field site can be found in the Photographs subfolder.

### The main November - December deployment

The GoPro data of both GoPros of the main November - December deployment are stored in the *20211109 20211215 Main deployment - GoPro 1 - Nearshore - RealDune/REFLEX* and *20211109 20211215 Main deployment - GoPro 2 - Nearshore - RealDune/REFLEX* data sets. The lidar data of the main November - December deployment are stored in the *20211109 20211215 Main deployment - lidar 1 - Nearshore - RealDune/REFLEX*, *20211109 20211215 Main deployment - lidar 2 - Nearshore - RealDune/REFLEX*, and *20211109 20211215 Main deployment - lidar 3 - Nearshore - RealDune/REFLEX* data sets for L1, L2 and L3, respectively. All remaining data of the main November - December deployment are stored in the data set *20211109 20211215 Main deployment - Nearshore - RealDune/REFLEX*.

The GPS data are divided into a folder for each day, with notation YYYYMMDD. The PS data are gathered in two netcdf files, one for the OSSI pressure sensors (*20211109 20211215 ossi.nc*) and one for the RBR pressure sensors (*20211109 20211215 rbr.nc*). The ADV and OBS data are stored in four netcdf files, with one file for each ADV. The OBS data of the OBSs at station S06 and S14 can be found in the netcdf of the ADV at those stations (*S06_ADV_02.nc* and *S14_ADV_04.nc*). The OBS data in the ADV netcdf files is given in counts. These values should be converted to volts, using the formula provided in the instrument manual. A copy of this formula is given in the *OBS calibration files* folder. The value in volts can be converted to g/l using a conversion diagram based on the results of OBS calibrations. This conversion curve is also stored in the *OBS calibration files* folder. The JFE folder contains two netcdf files. The first netcdf file includes one hour of velocimeter data from the afternoon of November 21 2021 (*202111210200 jfevsadv.nc*), which is used in the Technical Validation section to assess the consistency between horizontal velocities measured by the JFE and ADV. The second netcdf file includes velocimeter data recorded by each JFE deployed during the December storm (Fig. 7, *20211130 20211203jfe.nc*). Data from the lidars are stored in.mat files for each burst within the lidar subfolder. For each lidar (L1, L2, and L3), there is a raw folder with the raw instrument output, an intermediate folder with point clouds with respect to the lidar origin for each hourly burst, and a clean folder where the point clouds have been rotated and translated from the local lidar coordinate system into the Dutch ordnance system (RDNAP). In addition, the clean folders contain lidar point clouds in cross-shore and longshore coordinates. The lidars did not always produce data when they were in the field, most likely because the intensity of the reflected beam was insufficient, as well as power supply and/or technical issues. As a consequence, there are not always intermediate and clean data files for the deployment times of Fig. 7. There are only clean files for the bursts during the December storm (December 1 to 2), as this was the only event with significant morphodynamic change of the dune face during the main November - December deployment. There are no L2 clean files because L2 malfunctioned and no data were recorded during the December storm.

The instrument coordinates and heights above the bed are assembled in the *20211109 20211215 instrument heights.nc* and *20211109 20211215 instrument heights extrapolated xyz.nc* files. The vertical coordinates and instrument heights are given with respect to the instrument sensors. The z-coordinate and h-bed include an old (h_bed_old, z_old) and new (h_bed_new, z_new) value, in case the height of an instrument was adjusted during a survey. If both values are the same (*i.e*., h_bed_old = h_bed_new or z_old = z_new), the sensors were not moved vertically along the pole, whereas if the values are different, this indicates the distance the sensor was moved vertically. NaNs indicate (1) the instrument was not deployed in the field (see Fig. 7) or (2) the coordinates and/or elevations were not recorded during that specific survey. The first occurrence of h_bed_new and z_new indicates when the sensor was first deployed. On days when an instrument was removed from the field, the height prior to removal was recorded, if hydrodynamic conditions allowed, and the new value is NaN (*i.e*., h_bed_new = NaN), because the instruments were no longer deployed after the instrument height survey. When the elevation of an instrument was changed, the old or new z-location of the instrument was not always measured with the RTK GPS. This causes NaNs to be present in the coordinate values in the *20211109 20211215 instrument heights.nc* file before and/or after elevation changes. The file *20211109 20211215 instrument heights extrapolated xyz.nc* provides these coordinates, where the missing coordinates were computed using previous or next RTK GPS measurements, and documented changes in bed elevation. Additional information about this computation and the *20211109 20211215 instrument heights extrapolated xyz.nc* file can be found in the readme (*readme 20211109 20211215 instrument heights extrapolated xyz.txt*). The instrument coordinates of the lidar origins are not within the instrument heights netcdf but within the clean lidar .mat files.

The IR photographs from November 30 to December 4 are stored in the IR subfolder. The *Beach* folder contains photographs of the IR camera that faced the beach. The *Dune* folder contains photographs of the IR camera that faced the dune face. The GoPro photographs that were taken during the December storm are stored in the GoPro folder. Lastly, some general photographs of the field site and both dunes can be found in the Photographs subfolder.

It should be noted that during the second instalment of the RBRs at stations S01 and S11, between November 23 and December 4 (Fig. 7), the GPS coordinates of these two instruments could not be recorded due to strong hydrodynamic conditions. These coordinates can be acquired by using the height above the bed and the GPS transect measurement of November 24 (*20211124 TRANSECTS.txt*). A script to acquire these heights is in the raw log folder of the instrument heights folder (*extraction offshore stations.py*).

During the December storm, the frame at station 14 got tilted due to strong hydrodynamic conditions. As a consequence of this tilt, the vertical coordinates of the instruments at this station changed (1 ADV, 2 OBSs, and 1 RBR). The GPS instrument surveys of November 30 and December 3 can be used to compute the translation of the instruments due to this tilt. Vertically, it resulted in a translation of the instruments of approximately 7 cm.

### The January storm deployment

Data from the January storm deployment can be found in the *20220105 20220107 Storm deployment - Nearshore - RealDune/REFLEX* data set. The GPS data are divided into a folder for each day, with notation YYYYMMDD. The pre-storm GPS survey of January 5 is less detailed due to strong hydrodynamic conditions, which prevented measurements at deeper locations further seaward.

Photographs for an impression of the field site can be found in the Photographs subfolder. The PS data are gathered in a single netcdf file (*20220105 1400 20220107 1200 rbr.nc*). The processed data from the four JFEs are stored in one netcdf file. The L4 data files are stored in the lidar folder. For L4, there is a raw folder with the raw instrument output, an intermediate folder with point clouds with respect to the lidar origin for each hourly burst, and a clean folder where this point cloud has been rotated and translated towards the Dutch coordinate system (RDNAP). The instrument heights and coordinates are given in the instrument height file (*20220105 20220107 instrument heights.nc*). The elevations remained constant throughout the January storm deployment. Therefore, no distinction between an old or new value is made. The instrument coordinates of the lidar origin are only within the clean lidar.mat files.

### Supplementary data

The supplementary data set *Supplementary data - Nearshore - RealDune/REFLEX* contains records with the air pressure measured at the Hoek van Holland station of the KNMI (Royal Netherlands Meteorological Institute), and water levels recorded at Hoek van Holland and Scheveningen by Rijkswaterstaat (The Dutch Ministry of Infrastructure and Water Management). The air pressure files were obtained from https://www.knmi.nl/nederland-nu/klimatologie/uurgegevens. Water levels were obtained from https://waterinfo.rws.nl. The minimum mean water levels (*η*_min, RWS_) displayed in panels b) and c) in Figs. 5 and 8 are based on the Rijkswaterstaat water level measurements.

## Technical Validation

### Accuracy of the drone and jetski measurements

Topography measurements were conducted with a DJI Phantom 4 RTK drone on November 3 and December 17. Aerial images of the drone were converted to RDNAP coordinates through photogrammetry and Ground Control Points (GCPs) within the drone images. The RDNAP coordinates of the GCPs were measured with an RTK GPS and were used for georeferencing. In the intertidal zone, the topography data based on the drone was compared to and validated with measurements of an RTK GPS walking survey. For the November 3 survey, the mean vertical difference between the drone measurements and RTK GPS walking survey measurements was −0.011 m with a standard deviation of 0.073 m. The negative value means that the drone survey recorded elevations that were on average 0.011 m higher than the RTK GPS walking survey measurements. For the December 17 survey, the mean difference was −0.024 m with a standard deviation of 0.022 m.

Bathymetry measurements were conducted with a jetski on November 3, November 9, and December 13. The water depth below the jetski is measured with a Hydrobox Single Beam Echo Sounder (SBES), with a sampling frequency of 10 Hz. The water depth below the SBES is computed using the speed of sound in water and by computing the time difference between transmitting and receiving a signal. The position of the jetski in RDNAP coordinates is measured with an RTK GPS. The distance between the SBES and RTK GPS on the jetski is fixed throughout the survey. As a result, the water depth below the SBES can be used to compute the location of the bed in RDNAP coordinates. Additional information on the jetski measuring technique and the vertical and horizontal accuracy of jetski measurements can be found in van Son *et al*.^30^.

### Pressure sensors

Pressure was recorded by 6 RBR-, 8 OSSI-, and 4 ADV pressure sensors during the different deployments. During the main November - December deployment, the ADV pressure sensors at stations S01, S11 and S14 were accompanied by an RBR pressure sensor. The RBR pressure sensors logged the total pressure, including atmospheric pressure. The recorded pressure compared well to the air pressure measured at the Hoek van Holland station of the KNMI (Royal Netherlands Meteorological Institute). No significant offset or drift of pressure could be observed. The OSSI wave gauges and ADV pressure sensors had (1) a pressure offset due to a constant pre-defined atmospheric pressure and (2) a drift of this pressure offset with time.

For the initial November storm deployment, the pressure offset of the deployed OSSI was corrected for by using the air pressure at the Hoek van Holland station. The offset was based on the difference in recorded pressure by the OSSI and the air pressure at the Hoek van Holland station on November 6 between 21:00 and 23:58. The pressure offset was assumed to be constant over the initial November storm deployment. A potential drift of the offset was assumed to be negligible over the period of 2 days.

For the main November - December deployment, the pressure offset and pressure drift of the OSSIs were corrected for by using the air pressure just before the first instalment early November, and three bucket tests. The tests were executed on November 19, before the first service interval of the OSSIs and after the first instalment, November 20, after the first service interval and before the second instalment, and on December 14, after the second instalment (Fig. 7). During the bucket tests, the RBR and OSSI pressure sensors were dipped at the same time in a bucket of water with a constant water level.

The offset of each OSSI was based on the difference between the recorded pressure including offset by the OSSI, and (1) the recorded (actual) air pressure by the Hoek van Holland station, or (2) the recorded pressure by the RBRs during the bucket tests. The offset prior and after an instalment was not equal for the OSSIs, meaning there was a drift of the offset over the course of an instalment. S12_OSSI_07 experienced the largest drift of approximately 1200 Pa over the first instalment from November 9 to November 19, and 1700 Pa over the the second instalment from November 20 to December 12. The other OSSIs experienced drifts of approximately 100–500 Pa for both instalments. In the correction for the drift of the OSSIs a linear change of the offset over time was assumed.

The internal clocks of all OSSI and RBR pressure sensors were time-synchronised using a computer connected to internet time prior to deployment. The bucket tests were used to remove possible time-drifts of the instruments, and to time-synchronise all the different pressure sensors with each other. For this drift correction and time synchronisation, we assumed the time of S01_RBR_01 to be the correct time. We time-calibrated and synchronised all the other pressure sensors by having the dip peaks in the signals of all instruments coincide with the peak in the signal of S01_RBR_01 before and after the instalments. Overall, the other RBR pressure sensors displayed time drifts with respect to S01_RBR_01 smaller than 2 s for the main November-December deployment, and time drifts smaller than 0.7 s for the January deployment. The OSSI pressure sensors displayed larger time drifts with respect to S01_RBR_01. These drifts were of the order of 1 minute over the main November-December deployment. The final OSSI and RBR pressure sensor netcdfs are corrected for the pressure offsets, pressure drifts, and time drifts.

No dipping tests were performed with the ADV during the main November - December deployment. Therefore, the pressure offset and drift of the ADV pressure sensors were found by computing the difference between the pressure output of the ADV pressure sensor and the local air pressure measured at the Hoek van Holland station, before and after each instalment of an ADV (Fig. 7). Again, the pressure drift of the ADVs was assumed linear.

Lastly, during the initial November storm deployment and the main November - December deployment, the OSSI pressure sensors did not record the last 50 seconds of each day because they used that time to write the recorded data to a .csv file within the instrument. Therefore, the recorded pressure of the last 2 and first 2 minutes of each day were replaced by NaNs (Not a Number). The 50 second time interval was lengthened to 4 minutes to be conservative.

**Fig. 9:**
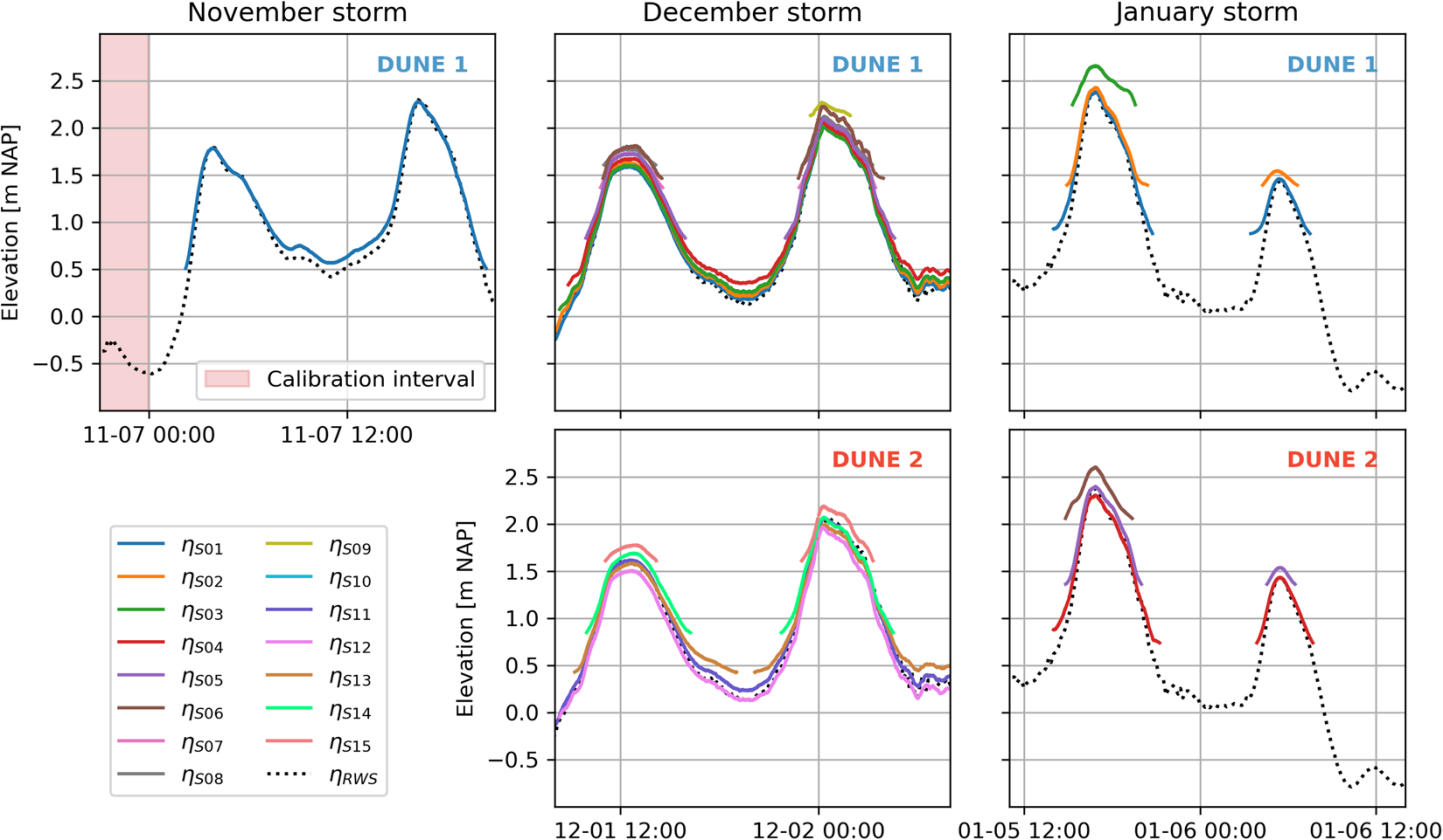
Water levels measured by the pressure sensors and an estimation of water levels at the Sand Engine based on data from Rijkswaterstaat (The Dutch Ministry of Infrastructure and Water Management) during the three storms. The interval used to compute the pressure offset of the OSSI during the initial November storm deployment is marked in red.

Figure 9 displays a comparison between the water levels based on the air pressure corrected and offset corrected pressure of the pressure sensors deployed in the field, and an estimation of the water levels recorded at the Sand Engine by Rijkswaterstaat (The Dutch Ministry of Infrastructure and Water Management). The Rijkswaterstaat estimation of the water levels at the Sand Engine is based on the water levels recorded at Scheveningen and Hoek van Holland. The Sand Engine is approximately in between these stations. The agreement between the time series is good, with small differences that can be attributed to local wave setup. Only during the December storm, the ADV pressure sensor at S06 displays a water level which is approximately 5–10 cm higher than the trend of the other pressure sensors.

### ADVs

**Fig. 10:**
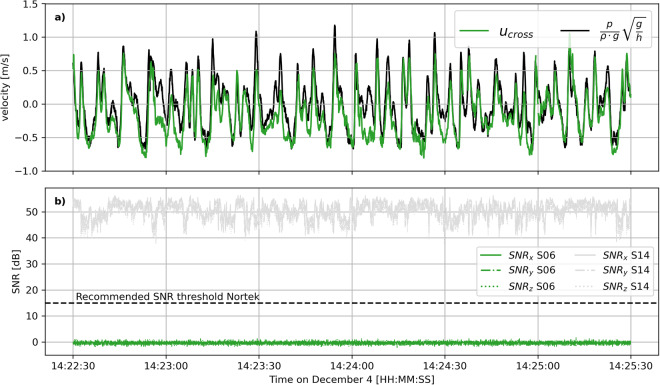
Quality check of the low signal-to-noise ratio (SNR) of S06_ADV_02 reported throughout the experiment. Panel (**a**) displays the cross-shore velocity signal *u*_*cross*_ (green) and the velocity signal based on converted co-located pressure measurements using shallow water linear wave theory (black). Panel (**b**) displays the SNRs for the velocity measurements in xyz direction of S06_ADV_02 (green). For reference, the SNRs for the velocity measurements of S14_ADV_04 during the same period in the same depth (∼1 meter) and at a similar elevation in the water column (0.4 versus 0.6 m below mean water level), are also displayed (grey). The instrument manufacturer Nortek recommends SNR higher than 15 dB when collecting raw data^24^.

The ADVs recorded pressure, velocity in its local coordinate system (XYZ), correlation, signal-to-noise, distance to the bed, temperature, and instrument orientation (i.e. heading, pitch, roll) data. All ADVs had the velocity probe fixed to the housing except for S01_ADV_01, which had the probe and housing connected through a cable. The velocity probes were deployed downward-looking with their X-leg pointing onshore along the shore-normal axis. The housing of S01_ADV_01 was mounted horizontally to the frame because of convenience, which, however, prevented the collection of valid orientation data. All ADVs recorded data successfully when deployed in the field. The variable *flag_data* in the netcdf files, indicating when the instrument was deployed, can be used to flag data that are not useful. Note that part of the data should still be removed after taking into account *flag_data*, because sensors were not always under water. Correlation and signal-to-noise data can be used to identify poor quality data. In particular, the ADVs located on the upper part of the beach (S06_ADV_02 and S14_ADV_04) were submerged intermittently. There was a constant low signal-to-noise ratio (<5 dB) reported by S06_ADV_02 throughout the experiment that did not vary based on instrument submergence. Based on follow up analysis, the backscatter intensity of this ADV was diagnosed to be always high. As a consequence, the noise floor is high too, resulting in low signal-to-noise ratios. However, validation of the velocity signal during the experiment with co-located pressure measurements showed that the velocity signal was not affected (Fig. 10). For this comparison the pressure signal was converted into a cross-shore velocity using shallow water linear wave theory, i.e. $u=\frac{p}{\rho g}\sqrt{\frac{g}{h}}$, where *p* is the recorded pressure, *ρ* the density of water, g the gravitational acceleration, and *h* the mean water depth (∼1 m in this case). Distance to the bed was recorded every hour using the bed ping option. However, most of the time the sensors were not located within the range required for bed ping measurements. The orientation of S06_ADV_02 changed substantially over the December storm because the cantilever on which the ADV was mounted turned around the pole of the station, whereas the orientation of S14_ADV_04 changed due to tilting of the frame. Also, the orientation of S01_ADV_01 changed substantially during the data collection period due to a turned cantilever but was only noticed when the instrument became visible during the low tide of December 6 when conditions were low energetic and the water level was particularly low (see Fig. 6d).

**Fig. 11:**
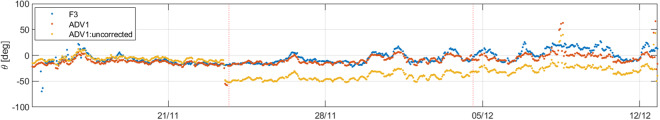
Energy-weighted wave angle at offshore station at 6 m depth (blue) and S01_ADV_01 before (yellow) and after (red) heading correction on 23 November 2021 and 4 December 2021.

The internal clocks of the ADVs were synchronised with the computer before each deployment. Time drift of the internal clocks over the data collection period differed between the instruments from 0.1 to 1.5 s. The ADV data set was not corrected for time drift. Raw velocity data in the ADV coordinate system (XYZ) were converted to a local coordinate system (cross-shore, longshore, up) and a global coordinate system (East, North, Up) using coordinate transformations with internally measured heading, pitch and roll. Hereto, heading, pitch and roll were averaged over intervals of 6 min, allowing to have stable orientation measurements that can account for persistent changes in ADV orientation (e.g. due to wave force). Orientation changes of S06_ADV_02 and S14_ADV_04 were accounted for in the coordinate transformation, but not of S01_ADV_01 because the lack of heading, pitch and roll data. Observations of S01_ADV_01 during the low tide of December 6 suggested that only heading, and not pitch and roll, changed substantially. The change in heading angle of the ADV was corrected through a directional analysis. First, the wave angle of S01_ADV_01 throughout the campaign was calculated assuming no change of the velocity probe position after ADV deployment. The energy-weighted mean angle was estimated through a principal component analysis on the horizontal velocity data following Herbers *et al*.^31^ and Henderson *et al*.^32^. The wave angle at S01_ADV_01 was compared to the wave angle measured ∼500 m offshore from the study site at ∼6 m depth. Figure 11 shows wave angles align until 23 November 2021. This moment the measurement station was serviced under challenging conditions. Probably, the cantilever got hit accidentally, and turned around the station pole. A heading correction of 32 degrees was applied in the coordinate transformation, leading to a wave angle at ADV1 that aligns with the other stations throughout the campaign. The directional analysis indicated that an additional correction of −9 degrees was needed from 4 December onwards.

### OBS calibration

The OBSs at stations S06 and S14 measured optical backscatter, which can be used as a proxy for suspended sediment concentrations. The measured optical backscatter is given in counts in the ADV netcdfs. The counts should first be converted to volts. After this, the conversion from volts to suspended sediment concentrations can be performed using the results of an OBS calibration performed by the University of Utrecht (UU). The calibration values and parameters are provided in the ADV + OBS folder of the main November - December deployment, in the OBS calibration files folder. The OBS calibration was performed in a closed pipe circuit at the UU, where the magnitude of suspended sediment concentrations could be controlled. Varying concentrations with sediments from the field site were replicated within the circuit. For each OBS, a calibration factor was acquired by comparing the variations in actual concentrations in the circuit with variations in instrument output.

For the OBSs at station S06 (serial numbers T9012 and T9205), a different gain factor was applied in the field than the one applied during instrument calibration. To account for this different gain factor, an additional conversion is necessary (see the readme in the OBS calibration files subfolder for more information). This conversion can be based on the instrument calibration forms after fabrication, provided to us by Campbell Scientific. These forms can also be found in the ADV + OBS folder. A conversion script has been added for convenience.

The OBSs at stations S06 and S14 were placed in close vertical proximity to each other to be able to compare measurements and gain confidence in the recorded data. Overall, at times when the OBSs were submerged, after applying the calibration curve and without any additional data filtering, the OBS data reveal averaged concentrations of 1–10 g/l, with standard deviations of 0–10 g/l. Sediment peaks of 120 g/l were observed, which are unrealistic and highlight the need for additional filtering.

### 2DV lidar Scanners

**Fig. 12:**
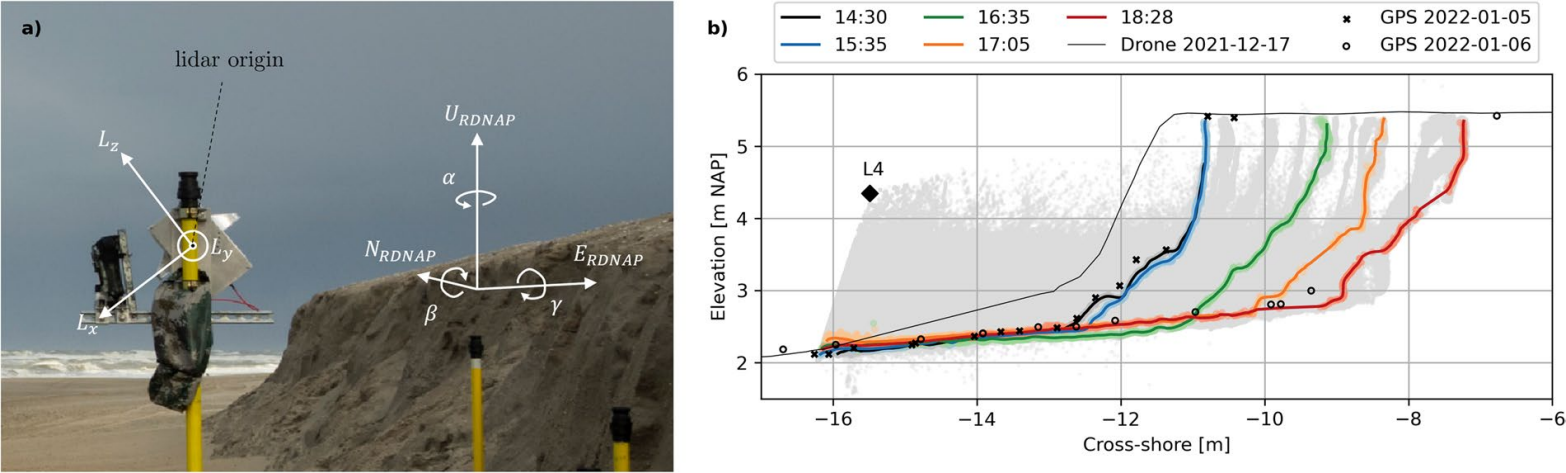
(**a**) Impression of the lidar setup in the field and its local lidar coordinate system. (**b**) lidar point clouds for the January 5 data set. The angles of rotation, *α*, *γ*, and *β*, were calibrated by comparing the rotated lidar point cloud in the RDNAP coordinate system with RTK GPS data points and drone survey data points. Small differences between the GPS and lidar profiles can most likely be attributed to the GPS data points not being exactly on the lidar line-scanning transect. For the January data set, *α* = 154°, *γ* = 0°, and *β* = 51.669° (Eq. 1).

The point clouds of the 2DV lidar scanner for the December and January storms were converted to Dutch RDNAP coordinates using the lidar origin location within the Dutch RDNAP coordinate system, and the 3D orientation of the instrument consisting of a yaw, pitch, and roll angle

1 $$\begin{array}{c}{\overrightarrow{P}}_{RDNAP}={R}_{tot}\cdot {\overrightarrow{P}}_{lidar}+{\overrightarrow{O}}_{RDNAP}\\ {R}_{tot}={R}_{yaw}\cdot {R}_{roll}\cdot {R}_{pitch}=\left[\begin{array}{ccc}\cos \alpha  & -\sin \alpha  & 0\\ \sin \alpha  & \cos \alpha  & 0\\ 0 & 0 & 1\end{array}\right]\cdot \left[\begin{array}{ccc}1 & 0 & 0\\ 0 & \cos \gamma  & -\sin \gamma \\ 0 & \sin \gamma  & \cos \gamma \end{array}\right]\cdot \left[\begin{array}{ccc}\cos \beta  & 0 & \sin \beta \\ 0 & 1 & 0\\ -\sin \beta  & 0 & \cos \beta \end{array}\right]\end{array}$$

where ${\overrightarrow{P}}_{RDNAP}$ is the point cloud in RDNAP coordinates, *R*_*tot*_ the rotation matrix consisting of yaw *R*_*yaw*_, roll *R*_*roll*_, and pitch *R*_*pitch*_ with angles *α*, *γ*, and *β*, respectively (Fig. 12), ${\overrightarrow{P}}_{lidar}$ the point cloud in lidar coordinates, and ${\overrightarrow{O}}_{RDNAP}$ the RDNAP coordinates of the lidar origin from which the beams of the instrument originate. A positive rotation is defined as clockwise while “looking” in the direction of the axis of rotation. The initial orientation stipulates that +*L*_*x*_ and +*L*_*y*_ are aligned east (*E*_*RDNAP*_) and north (*N*_*RDNAP*_), respectively, with the lidar puck origin at the global RDNAP origin.

The RDNAP coordinates of the lidar origin (center of the puck) were estimated based on the average values of RTK GPS measurements of the corners of the top of the lidar box, as well as known offsets from the center of the box top to the puck center. Yaw angle, *α*, was converted to compass heading angle via

2 $$Heading=9{0}^{\circ }-\alpha ,$$

where zero degrees heading is north, with heading angle increasing clockwise around *U*_*RDNAP*_. Lidar heading was assumed to be equal to the dune orientation for both the main November - December deployment and January storm deployment (heading = 296° true North). Roll angle, *γ*, was set to 0° for all data sets, as the pole to which the instrument was attached was levelled before deployment.

Initial estimates of the pitch angle, *β*, were based on the GPS measurements of the four corners on the top of the lidar box. The final value of *β* was calibrated by iterating through multiple values of *β* around the initial estimate, and by computing the total RMSE for each *β* between (1) The elevation of RTK GPS point measurements within the lidar transect, and (2) the elevation of the rotated lidar point cloud at the cross-shore coordinates of the RTK GPS point measurements. The GPS survey of December 10 was used for the main November - December deployment calibration, and the GPS survey of January 5 was used for the January storm deployment calibration. The minimized RMSEs between the elevation of the RTK GPS points and rotated lidar points were 0.029 m for L1, 0.024 m for L3, and 0.052 m for L4. There is no result of the calibration for L2 because no point clouds were measured at L2 during the time of GPS survey on December 10. After this calibration, *β* of L4 required an additional adjustment of −1.1°. This adjustment was based on the total January storm lidar point cloud, which had to remain within the December 17 profile cross-section of the drone-based SfM surveyed profile elevations (Fig. 12). The calibration procedures resulted in a *β* of 4.857° for L1, 40.415° for L3, and 51.669° for L4. Cross-shore and longshore lidar point cloud coordinates were computed by applying a 2D rotation of 26 degrees, about the Dune 1 local coordinate system origin, to the easting and northing Dutch RDNAP coordinates.

Additional information on the vertical and horizontal accuracy of the LLC lidar for measuring beach profiles, water levels, and the cross-shore extent of wave runup is described in detail in O’Connor and Mieras^27^. In their assessment, the LLC lidar did not contain a glass pane in front of the lidar puck, while the LLC lidar during RealDune/REFLEX was enclosed with a flat glass pane to protect the instrument against water. However, controlled tests indicated the radial distance to objects roughly 2 to 4 m away (similar distances from the lidars to the beach/dune in these experiments), through the glass pane versus no glass, differed by 0.001 to 0.003 m, which was deemed negligible for the scales of interest in dune erosion for this study.

**Fig. 13:**
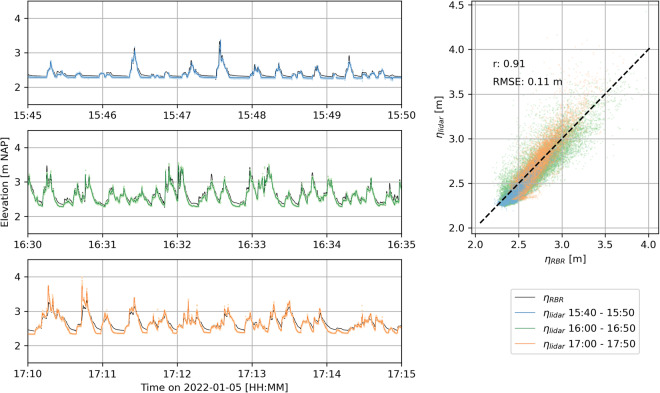
Comparison between water levels recorded by the RBR at S03 (Fig. 8) and lidar at the location of the RBR during the storm on January 5, 2022. The different panels display 5-minute segments of the lidar and RBR data sets between 15:40 and 17:50. The lidar data sets were shifted +3.271, +3.587, and +4.412 s for the 15:40, 16:00, and 17:00 bursts, respectively. r represents the Pearson’s product moment correlation coefficient.

The internal real-time clock (RTC) of each lidar instrument was synchronised with the local time before deployment using an internet time server while connected to WiFi. Still, the lidars experienced a small time drift throughout the deployment between the 35-minute (main November - December deployment) or 50-minute (January storm deployment) bursts, relative to other co-located instrumentation. The relative time offsets of each burst and each lidar were computed by comparing water levels between the pressure sensors and the lidar-derived water levels^27^ at the location of the pressure sensors (Fig. 13). The time offsets of the 15:40, 16:00, and 17:50 bursts on January 5 in Fig. 13 were 3.271, 3.587, and 4.412 s, respectively. These offsets were computed by minimising the RMSE between the timestamps of the largest 15 (15:40 burst) or 25 waves (16:00 and 17:00 bursts), based on the pressure sensor water levels and the lidar-derived water levels. Throughout the experiment, the time offsets between the lidars and co-located pressure sensors ranged between 0 and 10 s, with a mean of 1.322 s.

Such time synchronisations could only be performed during time spans when wave runup was within the range of both the lidar and co-located pressure sensor. Because this was not always the case, and to remain consistent in how the different lidar data files are published within this data set, none of the lidar data files have been corrected for time offsets.

### JFE 2DH Electromagnetic Current Meters

Electromagnetic current meters have been used previously to measure velocities in very shallow water–including the inner surf and swash zone^33–35^. These sensors can capture velocities near the bed and are less sensitive to bubbles, in contrast to ADVs, but are limited to horizontal velocities. The JFE 2DH velocimeters measure voltage–induced by water flowing through an electromagnetic field generated by the instrument–as well as instrument orientation.

Here, we convert voltage to horizontal velocities (easting and northing) using an empirical (linear) relationship provided by the manufacturer (based on benchmark tests in a controlled laboratory setting) and then rotate the velocities to match the local coordinate system using the measured (compass-derived) instrument orientation to obtain cross-shore (*u*) and alongshore (*v*) velocities. Each JFE was mounted to a cantilever, positioned approximately 25 cm from the vertical pole, with the sensor probe vertically pointed upwards (Fig. 7). At installation, each probe was positioned such that the measuring point was sufficiently above the bed (>10 cm), but the base of the probe was buried in sand (which ensures that the sensor remains grounded). The variable *flag_data* in the netcdf data files indicates measurements when the instantaneous measurement was at least 0.1 m below the time-dependent free surface and considered sufficiently submerged for analysis. The water level above the instantaneous measurement, as reported in the netcdf files, was calculated using atmospherically-corrected pressure measurements from the co-located pressure sensors and applying a hydrostatic water-surface reconstruction.

The internal clocks for each JFE were synchronised within 0.1 sec of internet time prior to deployment. Due to the relatively short deployments, which were limited by battery to less than three days, time drift was assumed to be small and therefore neglected. Any changes in the measured JFE orientation are adjusted for during processing of raw data (e.g., S10 with JFE04 rotated about 120 degrees during the Dec-02 storm). Rotations around the instrument *x*- and *y*-axis are not recorded and assumed to be constant throughout the deployments (i.e., assumed that sensors remain vertical). Thus, measurements cannot account for a tilting of the poles during their deployment.

**Fig. 14:**
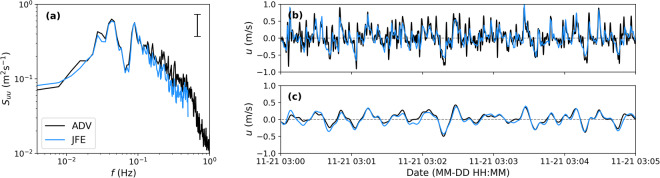
Cross-shore velocities (*u*) measured with a co-located ADV (black) and JFE (blue, adjusted for a 2.1 s lag behind the ADV) at S06 on November 21, 2021. (**a**) Cross-shore velocity spectra (*S*_*uu*_) as a function of frequency (*f*) with the 95% confidence interval. Cross-shore velocities time series (**b**) from 1 Hz JFE and 1 Hz low-passed ADV measurements and (**c**) 0.1 Hz low-passed JFE and ADV measurements.

A JFE velocimeter was co-located with an ADV (approximately 0.75 m horizontal separation) at S06 of Dune 1 in the afternoon of November 21 2021 to assess the consistency of horizontal velocities measured by both instruments (Fig. 14). The JFE sampled at 1 Hz with the measuring point positioned 50 cm above the bed. The ADV sampled at 16 Hz with the measuring point positioned 67 cm above the bed. Bed level changes during the sampling period, which spanned approximately 11 hours, were between 8 and 11 cm proximate to both instruments. During post-processing, the JFE time series were found to lag the ADV time series by 2.1 s, which is likely due to internal clock calibration to different computers before deployment (the JFEs were calibrated within 0.1 s of internet time whereas the ADV was not), and time drift in the ADV (deployed one week prior on November 13, Fig. 7).

We first assess low-frequency velocity measurement consistency between a co-located JFE and ADV. The 1 Hz JFE velocities (adjusted for a 2.1 s time lag) are similar to 1 Hz low-passed ADV velocities with a Butterworth filter (Fig. 14b, shown only for cross-shore velocities). When both data streams are filtered to even lower frequencies (using a Butterworth filter with a cutoff frequency of 0.1 Hz), the JFE and ADV cross-shore velocities are nearly identical (Fig. 14c). Auto-spectra from the cross-shore velocities (*S*_*uu*_) were computed using a Hanning window period of 256 s with an overlap period of 128 s for a 60 min time series (with 51 degrees of freedom, Fig. 14a). The spectra from the JFE and ADV are similar for frequencies less than 0.16 Hz, where differences between curves are within the 95% confidence interval. The ADV measures higher variance above 0.16 Hz than the JFE. Discrepancies between the JFE and ADV are possibly attributed to the lower JFE sampling frequency, different measurement positions in the water column (apart by 0.75 m horizontally and 17 cm vertically). Note that due to the low ADV signal-to-noise ratio, we cannot validate the JFE with the ADV, but rather the good agreement between the two sensors gives us confidence that JFE observations are valuable to assess low-frequency velocities (*f* < 0.16 Hz).

**Fig. 15:**
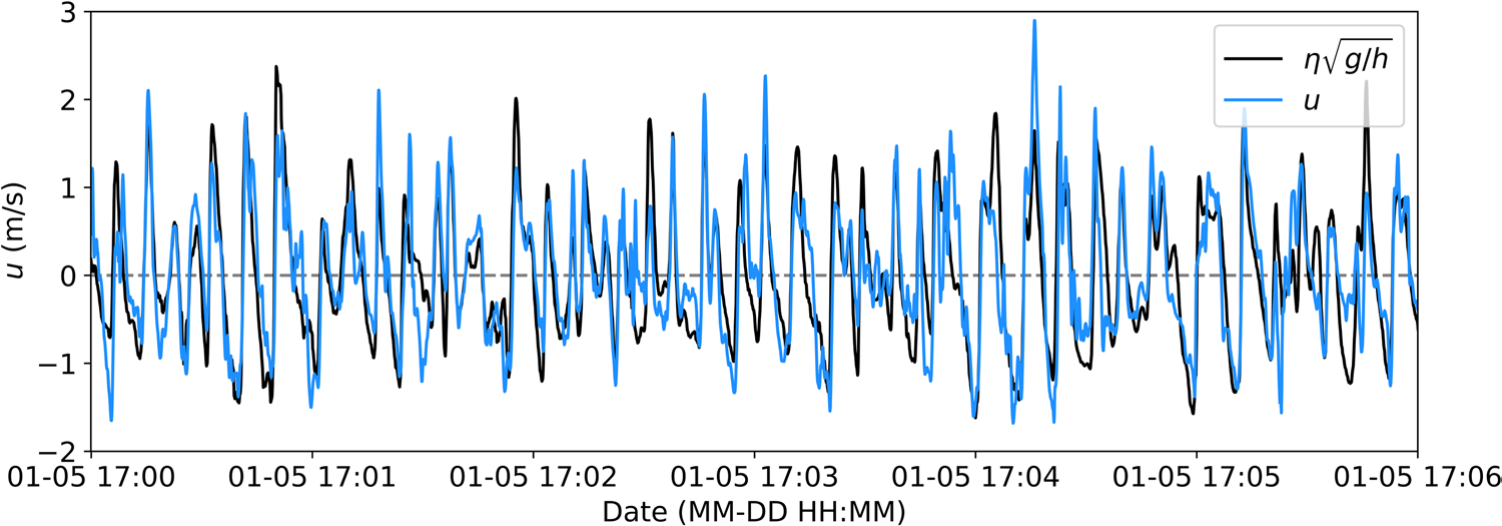
High-passed (*f* > 0.04 Hz) time series of cross-shore velocities (*u*) at S04 measured on January 5, 2022, with a JFE (blue, adjusted for a 1 s lag behind the pressure sensor) and estimated from pressure sensor measurements (black) converted to equivalent velocity using the linear dispersion relationship assuming shallow water ($\eta \sqrt{g/h}$).

During the January 5 event, the JFEs sampled at a higher frequency (10 Hz), however, the consistency between ADV and JFE horizontal velocity measurements at this sampling rate cannot be assessed because there were no co-located instruments during this storm event. Therefore, to assess the quality of the measured 10 Hz JFE velocities during this event, we compare JFE velocities with measurements from a co-located pressure sensor at S04 on January 5 (Fig. 8). The atmosphere- and depth-attenuation corrected pressure was converted to an equivalent velocity using the linear dispersion relationship, assuming shallow water, i.e. $\eta \sqrt{g/h}$, where *η* is the free surface elevation, *h* is the water depth, and *g* is gravitational acceleration. Again, a time lag was observed between the data streams–the JFE lagged the pressure sensor by 1 sec–which we attribute to internal clock drift. High-passed velocities (generated using a Butterworth filter with a cutoff frequency of 0.04 Hz) measured with a JFE at S04 and approximated from a pressure sensor at S04 are similar at surface gravity frequencies (*f* > 0.04 Hz), which gives credence to the quality of the JFE data and its ability to capture higher wave velocities when sampling at 10 Hz. Potential discrepancies between the two data streams are likely due to nonlinearities in the inner surf and swash zones (Fig. 15).

## Usage Notes

Usage notes are included in all relevant folders of the main data directory as *readme.txt* files. For all questions left unanswered concerning the data set, we recommend to contact the authors.

The nearshore measurements from October 2021 to January 2022 described in this paper were part of the RealDune/REFLEX field experiments, a larger framework of experiments that took place along the Dutch coast in the autumn, winter and spring of 2021/2022. Within these experiments, high-resolution hydrodynamic data were also collected from November 2021 to April 2022 at several offshore locations in close proximity to the two dunes. More details of these offshore measurements are described in an accompanying paper by Rutten *et al*.^12^. When the data set of Rutten *et al*.^12^ is combined with the data set of this paper, users have the opportunity to e.g. study offshore to nearshore wave transformation, link offshore wave conditions to nearshore morphodynamic (dune) response, or use actual wave boundary conditions in numerical models with the aim of replicating the erosion events of this study.

## Data Availability

Codes used in processing the data are included in the raw instrument folders.

## References

[1] Vellinga, P. duinafslag ten gevolge van de stormvloed op 3 januari 1976; toetsing van de voorlopige richtlijn. Tech. Rep., Waterbouwkundig Laboratorium, Delft (1978).

[2] Castelle B (2015). Impact of the winter 2013-2014 series of severe Western Europe storms on a double-barred sandy coast: Beach and dune erosion and megacusp embayments. Geomorphology.

[3] Masselink G (2016). The extreme 2013/2014 winter storms: Hydrodynamic forcing and coastal response along the southwest coast of England. Earth Surface Processes and Landforms.

[4] Hinkel J (2013). A global analysis of erosion of sandy beaches and sea-level rise: An application of DIVA. Global and Planetary Change.

[5] Ranasinghe R (2016). Assessing climate change impacts on open sandy coasts: A review. Earth-Science Reviews.

[6] Larson, M. & Kraus, N. C. SBEACH: numerical model for simulating storm-induced beach change; report 1: empirical foundation and model development. Tech. Rep., US Army Coastal Engineering Research Center (1989).

[7] Steetzel, H. J. *Cross-Shore Transport during Storm Surges*. Ph.D. thesis, Delft University of Technology. 10.1061/9780872627765.147 (1993).

[8] van Gent MR, van Thiel de Vries JS, Coeveld EM, de Vroeg JH, van de Graaff J (2008). Large-scale dune erosion tests to study the influence of wave periods. Coastal Engineering.

[9] Roelvink D (2009). Modelling storm impacts on beaches, dunes and barrier islands. Coastal Engineering.

[10] Den Heijer, C. *The role of bathymetry, wave obliquity and coastal curvature in dune erosion prediction*. Ph.D. thesis, Delft University of Technology. 10.4233/uuid:824df068-8046-414c-a1cc-7d159718918e (2013).

[11] van Wiechen P, de Vries S, Reniers A, Aarninkhof S (2023). Dune erosion during storm surges: A review of the observations, physics and modelling of the collision regime. Coastal Engineering.

[12] Rutten, J. *et al*. Continuous wave measurements collected in intermediate depth throughout the north sea storm season during the realdune/reflex experiments. 10.20944/preprints202403.0772.v1, Preprint at https://www.preprints.org/manuscript/202403.0772 (2024).

[13] Stive MJ (2013). A New Alternative to Saving Our Beaches from Sea-Level Rise: The Sand Engine. Journal of Coastal Research.

[14] de Schipper MA (2016). Initial spreading of a mega feeder nourishment: Observations of the Sand Engine pilot project. Coastal Engineering.

[15] Rutten J, Dubarbier B, Price TD, Ruessink BG, Castelle B (2019). Alongshore Variability in Crescentic Sandbar Patterns at a Strongly Curved Coast. Journal of Geophysical Research: Earth Surface.

[16] Roest B, de Vries S, de Schipper M, Aarninkhof S (2021). Observed changes of a mega feeder nourishment in a coastal cell: Five years of sand engine morphodynamics. Journal of Marine Science and Engineering.

[17] Watermanagementcentrum Nederland. Stormvloedflits 2022-01 Van 4 en 5 januari 2022. Tech. Rep., Rijkswaterstaat (2022).

[18] Vellinga, P. *Beach and Dune Erosion during Storm Surges*. Ph.D. thesis, Delft University of Technology (1986).

[19] van Thiel de Vries JS, van Gent MR, Walstra DJ, Reniers AJ (2008). Analysis of dune erosion processes in large-scale flume experiments. Coastal Engineering.

[20] Sallenger J (2000). Storm impact scale for barrier islands. Journal of Coastal Research.

[21] Leica. Leica Viva GNSS GS14 ontvanger Datasheet. Tech. Rep., Leica (2023).

[22] Ocean Sensor Systems. OSSI-010-003 Wave Gauge User Manual. Tech. Rep., Ocean Sensor Systems (2023).

[23] RBR Global. RBR solo and RBR duet Instrument Guide. Tech. Rep., RBR Global (2022).

[24] Nortek. Vector (ADV) Manual. Tech. Rep., Nortek (2018).

[25] Campbell Scientific Inc. Operator’s Manual OBS-3 + Suspended Solids and Turbidity Monitor. Tech. Rep., Campbell Scientific Inc. (2008).

[26] JFE Advantech Co. Ltd. Infinity electromagnetic current meter. Tech. Rep., JFE Advantech Co. Ltd. (2023).

[27] O’Connor, C. S. & Mieras, R. S. Beach Profile, Water Level, and Wave Runup Measurements Using a Standalone Line-Scanning, Low-Cost (LLC) LiDAR System. *Remote Sensing* **14**, 10.3390/rs14194968 (2022).

[28] Quartel S, Kroon A, Ruessink BG (2008). Seasonal accretion and erosion patterns of a microtidal sandy beach. Marine Geology.

[29] van Wiechen, P. *et al*. Nearshore coastal measurements of calm, moderate, and storm conditions at two artificial dunes along the Dutch Coast during the RealDune/REFLEX experiments. *4TU.ResearchData*10.4121/0a05d041-00b6-4e8e-a5c5-70e624ea720b (2023).

[30] Van Son STJ, Lindenbergh RC, De Schipper MA, De Vries S, Duijnmayer K (2010). Application Monitoring bathymetric changes at storm scale technical. PositionIT.

[31] Herbers THC, Elgar S, Guza RT (1999). Directional spreading of waves in the nearshore. Journal of Geophysical Research: Oceans.

[32] Henderson SM, Guza RT, Elgar S, Herbers THC (2006). Refraction of Surface Gravity Waves by Shear Waves. Journal of Physical Oceanography.

[33] Butt T, Russell P, Puleo J, Miles J, Masselink G (2004). The influence of bore turbulence on sediment transport in the swash and inner surf zones. Continental Shelf Research.

[34] Masselink G, Russell P, Turner I, Blenkinsopp C (2009). Net sediment transport and morphological change in the swash zone of a high-energy sandy beach from swash event to tidal cycle time scales. Marine Geology.

[35] Puleo JA, Cristaudo D, Torres-Freyermuth A, Masselink G, Shi F (2020). The role of alongshore flows on inner surf and swash zone hydrodynamics on a dissipative beach. Continental Shelf Research.

